# High-Coverage Whole-Exome Sequencing Identifies Candidate Genes for Suicide in Victims with Major Depressive Disorder

**DOI:** 10.1038/s41598-017-06522-3

**Published:** 2017-08-02

**Authors:** Dóra Tombácz, Zoltán Maróti, Tibor Kalmár, Zsolt Csabai, Zsolt Balázs, Shinichi Takahashi, Miklós Palkovits, Michael Snyder, Zsolt Boldogkői

**Affiliations:** 1 Department of Medical Biology, Faculty of Medicine, University of Szeged, Somogyi B. u. 4., Szeged, H-6720 Hungary; 2 Department of Genetics, School of Medicine, Stanford University, 300 Pasteur Dr., Stanford, CA 94305-5120 USA; 3 Department of Paediatrics, Faculty of Medicine, University of Szeged, Korányi fasor 14-15., Szeged, H-6720 Hungary; 4 Neuromorphological and Neuroendocrine Research Laboratory, Department of Anatomy, Histology and Embryology, Semmelweis University, Budapest, Üllői u. 26., H-1085 Hungary

## Abstract

We carried out whole-exome ultra-high throughput sequencing in brain samples of suicide victims who had suffered from major depressive disorder and control subjects who had died from other causes. This study aimed to reveal the selective accumulation of rare variants in the coding and the UTR sequences within the genes of suicide victims. We also analysed the potential effect of STR and CNV variations, as well as the infection of the brain with neurovirulent viruses in this behavioural disorder. As a result, we have identified several candidate genes, among others three calcium channel genes that may potentially contribute to completed suicide. We also explored the potential implication of the TGF-β signalling pathway in the pathogenesis of suicidal behaviour. To our best knowledge, this is the first study that uses whole-exome sequencing for the investigation of suicide.

## Introduction

Close to 20 million suicides are attempted annually worldwide, of which more than one million are completed^1^. Suicide is the 10^th^ leading cause of mortality in the world, which supports the importance of better defining the genetic causes and social basis of this disorder, and to identify individuals at risk. Suicide is a complex behaviour, determined by the interaction between proximal and distant risk factors. The proximal factors include recent life events, substance abuse and mental disorders, such as major depressive disorder (MDD), bipolar disorder and schizophrenia. The most important distal factors are the genetic and epigenetic factors, family history, early-life adversity and personality^2, 3^ The most common underlying disorder is MDD, which is the leading cause of disability worldwide^4^; more than 50% of suicide victims suffer from this disease, which increases the risk of suicide by up to twentyfold^5^. A number of studies have shown a familial accumulation of suicidal behaviour including suicide completion and attempt^6^.Twin and adoption studies have revealed that the heritability of suicide ranges between 30–55%^7^.

According to the current consensus, depression is etiologically a heterogeneous disease with overlapping causal pathways^8^, but logically, completed suicide with MDD may have a much less diverse genetic background. The heritable components of suicidal behaviour have until recently only been investigated either by hypothesis-driven research that focuses on preselected candidate genes^9–11^, or by the comparison of the frequencies of common genetic variants^12, 13^. Neurobiological evidence implicates the dysfunction of the HPA axis^14, 15^, as well as the serotonergic^16–18^, the dopaminergic^19, 20^ and other systems in suicidality.

The candidate gene approach has to date yielded very few results with general consensus. Genome-wide association studies (GWASs), in spite of their large sample sizes have not explored any association signals in depression^21, 22^, which may be in connection with the heterogeneous genetic background of MDD or it may also be possible that the causative genetic factors of depression could lie outside of the scope of these studies. In contrast to candidate gene and GWASs, whole-exome studies (WES) or whole-genome studies (WGS) allow for the comparison of genomes with base pair precision, and are therefore capable of revealing rare genetic variants that potentially play a causative role in suicidal behaviour. Additionally, WES and WGS techniques allow the examination of the potential pathogenic role of expansion of short tandem repeats (STRs), copy number variations (CNV) and infection by viruses.

Until recently, the common disease-common variant hypothesis has been the ruling concept, and has been the theoretical basis for GWAS. However, it turns out that common alleles can explain only a fraction of the heritability of common diseases^23^. Today, we are witnessing the emergence of the common disease–rare variant hypothesis^24^, which proposes that rare variants may also be an underlying factor in common diseases, meaning that the same common disease can have different rare causative variants in different individuals. WES analysis is able to identify novel rare genetic variants, as well as common variants associated with monogenic^25^ and complex diseases^26^. WES platforms can be especially successful in the identification of heterogeneous single-gene disorders (umbrella diseases), which can be caused by multiple high-penetrance rare genetic factors.

In principle, suicide may be a heterogeneous Mendelian phenotype or, it may also be possible that genes that are known to cause single gene disorders may also confer risk of suicide in a certain percentage of patients. Indeed, an investigation of the medical records of 110 million patients demonstrated an association between monogenic disorders and complex diseases, such as MDD, bipolar disorder, and schizophrenia^27^.

In this study, we applied an Illumina HighSeq platform-based high-coverage WES technique, which, in addition to the exons, allows the determination of 5′- and 3′-UTRs, promoters to a certain length, along with off-target sequences, such as introns, intergenic regions and infecting viruses. The limitation of the WES platform is that it is not able to study potential factors located in the inter-genomic or deep intronic regions. However, it is estimated that 85% of penetrant disease-causing mutations reside in the coding regions of the genome^28^; therefore WES analysis has the potential to uncover the causes of rare variants of both homogeneous and heterogeneous monogenic disorders. In our study, all suicide victims suffered from MDD. We analysed the Hungarian population, which is known to have a high incidence of suicide^29^. To our best knowledge, until now no WES platform has been used for studying suicide, but a low-coverage WGS study on MDD has recently been published^30^.

## Results

### Analysis of biallelic variants

Based on the hypothesized molecular basis (risk/protective alleles and rare variants), we carried out specific bioinformatic analyses to investigate the potential genetic factors contributing to suicide. We assumed that within the suicide cohort the random distribution of high impact (HI) mutations throughout a gene represents loss-of-function variants, whereas an accumulation of mutations at specific regions within a gene is supposed to indicate a gain-of-function mechanism through altering the operation of protein domains or regulatory sequences. We assumed that genes having HI common variants with MAF higher than the incidence of suicide cannot be accountable in the loss-of-function model, and so we excluded them from the analysis.

### Analysis of region-specific accumulation of rare genetic variants

Gene function can be changed either by domain-specific mutations of the exonic regions resulting in altered protein function, or by mutations in the regulatory motifs that could lead to abnormal level or pattern of gene expression. Contrary to the loss-of-function mutations, which can be randomly distributed along the entire gene, gain-of-function mutations are supposed to be localized in well-defined regions. In the examined 594,910 genomic regions we identified 14,393 rare (AF < 1/5,000) variants that were not present in the Hungarian controls. The vast majority of the genomic regions contained no rare variants, 13,459 regions had only a single rare variant, in 808 regions 2, in 101 regions 3, and in 21 regions, at least 4 suicide samples had rare variants in the same genomic region (Table 1).

**Table 1:** Genomic region-dependent accumulation of rare variants in suicide samples.

SAMPLE COUNT	NUMBER OF VARIANTS	GENOMIC RANGE	GENE	REGION	SUICIDE SAMPLE ID
7	1	19:535931-536148	CDC34*	UPSTREAM	Y482,Y375,Y393,Y426,Y532,Y787,Y919
6	1	5:111091613-111091736	NREP*	SPLICE	BIB82,Y316,Y331,Y482,Y516,Y558
6	4	2:242666998-242668839	ING5*	3' UTR	Y166,Y292,Y591,Y724,Y988,Y787
5	4	11:117156433-117160295	BACE1	3' UTR	BrA206,Y331,Y645,Y919,Y292
5	2	22:23505688-23506663	RAB36	3' UTR	Br333,Y331,Br857,Y166,Y558
5	5	1:118507671-118509205	SPAG17*	3' UTR	Y375,Y393,Y421,Y520,Y532
5	5	2:240504919-240507676	ENST00000358775*	3' UTR	BIB82,Y375,Y426,Y532,Y724
4	4	X:17750592-17754159	NHS	3' UTR	Y426,Y516,Y532,Y919
4	1	15:20875075-20875135	NBEAP1*	INTRON	BIB82,BrA206,Y724,Y919
4	1	5:147649533-147649718	SPINK13	INTRON	Y331,Y393,Y532,Y591
4	1	20:2732322-2732689	EBF4	UPSTREAM	BIB82,Y393,Y516,Y558
4	2	3:130282083-130282518	COL6A6*	EXONIC	Y166,Y292,Y724,Y482
4	2	12:42475627-42481688	GXYLT1*	3' UTR	Y166,Y591,Y919,Y787
4	3	2:210884434-210885839	RPE	3' UTR	Br857,Y919,Y558,Y591
4	3	7:139246399-139257490	HIPK2*	3' UTR	Y166,Y787,Y292,Y516
4	3	9:87486857-87492822	NTRK2	3' UTR	Br333,Y375,Y426,Y645
4	4	18:11852188-11853806	GNAL	3' UTR	Br857,Y292,Y421,Y787
4	4	1:3350379-3355244	PRDM16	3' UTR	Y292,Y421,Y426,Y988
4	4	7:141356464-141362505	KIAA1147*	3' UTR	Br333,Y292,Y375,Y645
4	4	4:164445811-164449937	MARCH1*	3' UTR	Y166,Y516,Y532,Y988
4	4	15:67482895-67487583	SMAD3	3' UTR	Br857,BrA206,Y421,Y532

This table lists the genomic regions in which at least 4 suicide samples had rare variant(s). The SAMPLE COUNT column contains the number of suicide victims with rare variants in the same gene, the NUMBER OF VARIANTS column contains the number of individual variants within the gene, the GENOMIC RANGE column shows the GRCh37 start/end coordinates of the region, the GENE column contains the name of the gene, the REGION column contains the type of the genomic region, the SAMPLE ID column contains the suicide sample IDs with the rare variant in the given genomic regions.

*denotes genes without disease association in MalaCards and OMIM.

We identified a single exonic location (in COL6A6 gene encoding the alpha 6 chain of collagen type VI, which plays a role in axon guidance) that accumulated rare variants in more than 17% of the suicide cohort. Intriguingly, COL6A6 was also identified as a candidate gene in our recessive loss-of-function analysis (see below). Among the related pathways of this cell-binding protein are the interleukin and GM-CSF signalling and NCAM1 interactions^31^.

Additionally, we identified 20 non-exonic (putative regulatory) regions that contained rare variants in ~20% of suicide patients, which may contribute to the completed suicide. We must note that some of the variants in the list may be population-specific low MAF variants that were obtained because of the high number (594,910) of genomic regions examined in this study and the random selection of the individuals. The GNAL gene has been shown to contribute to schizophrenia^32^, the BACE1 gene is a candidate for Alzheimer’s disease^33^, while the NREP gene is associated with neural regeneration. In this analysis, we observed the highest enrichment in the CDC34 with 7 sample counts; this gene encodes an ubiquitin-conjugating enzyme playing a role in the control of cell cycle^34^. For the detailed list of the 64 individual variants, see Table S1.

Together, this part of our study revealed that there is no such gene in our cohort that could alone be accountable for causing suicide by domain-specific exonic alterations, but some rare alleles may play a non-exclusive causative role in this disorder.

### Identification of putative dominant loss-of-function rare variants

We identified 61 different protein damaging mutations (19 frameshift, 21 splice site, 3 start-loss, 5 stop-loss, and 13 stop-gain mutations), which were found exclusively in the suicide patients (Table 2). Only a single HI mutation (stop-loss, gene MRPL45) was present in two suicide individuals. MRPL45, encoding a component of the large subunit of the mitochondrial ribosome, is a Y-linked gene that if truly proves to be a risk factor, may explain the higher incidence of males for committing suicide.

**Table 2:** Putative dominant loss-of-function candidate genes where at least one sample had a rare protein disruptive variant in the suicide samples.

GENE	CHR	POS	REF	ALT	CONSEQUENCE	SAMPLE ID
MRPL45*	17	36478478	A	C	stop-loss	BrA206,Y426
CACNA2D4	12	2024019	C	A	splice donor & intron	Y516
OTOGL	12	80764471	T	C	splice donor & intron	Y591
CWC27	5	64084836	C	T	stop-gain	Y520
SPATA31C2*	9	90748568	T	A	splice acceptor & intron	Y558
RGL4*	22	24041028	CAGCTACAAGCTGT	C	splice acceptor & splice region & intron & non-coding transcript exon	Y331
PMCH	12	102591506	AAGTT	A	frameshift	Y532
TRIM15*	6	30131613	CG	C	frameshift	Br333
CEP85L	6	118880200	T	G	stop-gain	Y166
NME1	17	49231585	G	C	splice acceptor & intron	Y166
ZNF718*	4	60294	T	C	stop-loss & splice region	Y375
CENPC	4	68385221	C	G	splice acceptor & intron	Y724
CDK14*	7	90338857	T	C	start-loss	Y591
RAB3GAP2	1	220363490	T	C	splice acceptor & intron	Y292
PMM2	16	8900171	A	G	splice acceptor & intron	Y375
TMPRSS11F*	4	68919659	G	A	stop-gain	Y919
SURF4*	9	136233553	T	A	splice acceptor & intron	Y482
CKB	14	103988441	G	A	stop-gain	Y919
PCYOX1L*	5	148742545	CAA	C	frameshift	Y532
MIXL1	1	226413300	T	A	stop-gain	Y516
D2HGDH	2	242688420	CCCTGTGAGGATGGT	C	splice donor & splice region & intron	Y724
PSKH1*	16	67961717	C	T	splice acceptor & intron	Y316
BRMS1L	14	36333074	A	G	splice acceptor & intron	Y919
ST14	11	130058476	AT	A	frameshift	Y591
STRA8*	7	134925307	CA	C	frameshift	Y919
ARRDC2*	19	18120687	C	T	stop-gain	Y292
BOD1L1*	4	13629016	GC	G	frameshift	Y292
H2AFZ*	4	100871387	T	C	splice donor & intron	Y426
CFAP70*	10	75056798	A	G	splice donor & intron	Y166
SKOR2*	18	44746383	T	C	splice acceptor & intron	Y558
PRKAG2	7	151573704	A	G	start-loss	Y316
ADRA1A	8	26636945	C	A	stop-gain & splice region	Y516
TMEM132C*	12	129189800	G	T	stop gain	Y421
S100A13*	1	153600595	A	AC	splice donor & intron	Y724
DLG2	11	83191415	G	A	stop-gain & splice region	Y331
PCSK5	9	78790138	AAATGGAATGGAATGAAATGGAATGGAATGGAATGG	A	frameshift	Y421
C1orf226*	1	162353052	CCA	C	frameshift	Y426
EPS15	1	51946947	GTC	G	frameshift	Y482
WDR12*	2	203749260	T	C	splice acceptor & intron	Y375
TMA16*	4	164415989	G	A	splice donor & intron	Y645
LOC100507443*	2	208993176	C	CA	frameshift	Y516
CES4A*	16	67035297	TC	T	frameshift	Y558
NUDCD3*	7	44530037	G	GCT	frameshift	Y724
TP53RK	20	45315393	T	C	stop-loss	Y331
RABGGTA	14	24737761	C	T	stop-gain	Y919
UBE2E3*	2	181846846	AC	A	frameshift	Y166
MPDZ	9	13140066	A	G	start-loss	Br857
TNFRSF11B	8	119936822	G	A	stop-gain	Y919
ABI1	10	27054244	TC	T	frameshift & splice region	Y919
HAPLN1	5	82940440	TGA	T	frameshift	BrA206
MRAP2	6	84772679	C	CT	frameshift	Y591
CAND2*	3	12854548	G	T	stop-gain	Y393
CACNA1C	12	2659708	A	C	splice acceptor & intron	Y988
KRTAP2-4*	17	39221826	G	GA	frameshift	BrA206
SORL1	11	121502724	G	C	splice acceptor & intron	Y520
LRRC37A4P*	17	43585907	T	C	splice acceptor & intron	Y482
PEMT	17	17409148	T	G	stop-loss	Y645
RBMXL2	11	7110854	GC	G	frameshift	Br333
ZNF646*	16	31091705	C	T	stop-gain	Br857
RBM12B*	8	94752787	T	A	stop-loss	Y787
TGIF2LY*	Y	3447816	GCC	G	frameshift	Y645

Each genetic variant was a heterozygote. GENE is the name of gene; CHR and POS shows the GRCh37 coordinate of the variant, REF/ALT columns shows the reference and alternate alleles, CONSEQUENCE is the type of the protein disruption, and the SAMPLE ID column contains the sample names in which the given variant was found.

*denotes genes without disease association in MalaCards and OMIM.

From this cohort, 31 had no disease association in MalaCards and OMIM (Table 2). We found a male specific (Y chromosome) HI mutation in the TGIF2LY gene, too. Among the remaining 30 disease-associated genes, eight genes have been shown to contribute to neurological disorders. Note that CACNA1C encode a calcium ion channel, such as we found in the analysis of common variant risk factors (CACNA1B). Intriguingly, a genome-wide analysis has also revealed that CACNA1B along with the CACNA1A gene play a shared effect on 5 major psychiatric disorders, including MDD^35^. In this part of our study, we identified another calcium channel protein, CACNA2D4, which has been shown to play a role in the pathogenesis of bipolar disorder^36^.

In the second part of this study, we broadened our scope by including MI variants in the analysis. We selected genes in which at least two suicide samples had a rare variant (AF < 1/5,000 in databases; and so, it is not found in the Hungarian controls). We identified altogether 42 genes with 86 possibly protein damaging mutations found exclusively in the individuals who committed suicide (Table 3). The DOT1L and TTC28 genes were detected in four samples. None of these genes has been associated with any diseases so far. For five genes (TTC34, SCLY, SPHKAP, SOGA1, YES1) we found MI rare variants in 3 samples, all the other genes had only MI mutations in two samples (Table 3). In this cohort, seven genes have already been associated with neurological disorders (MNX1, NINJ1, PER2, PHF20, PRSS56, RPH3A, and SBF1) in MalaCards and OMIM. See the detailed list containing information about the individual variants in Table S2.

**Table 3:** List of putative dominant loss of function candidate genes where at least two samples had rare potentially damaging variants exclusively in the suicide samples.

GENE	SUICIDE SAMPLE COUNT
DOT1L*, TTC28*	4
TTC34*, SCLY*, SPHKAP*, SOGA1*, YES1	3
OTOG, PIK3R4*, SHISA6*, ZBTB49*, TATDN2*, DEDD2*, MAP3K14-AS1*, SMARCC1*, TXLNA*, DGKA*, C1QTNF7*, ZFC3H1*, BIK*, STC2*, GMEB2*, KLF7*,APLP2*, DCC, TNS1, PER2, MIXL1, KIAA1429, MCAM, LAMA3, MNX1, PRSS56, KIAA1549, SBF1, MRPL45, MYBPC1, PHF20, VAC14, RPH3A, NINJ1, REST	2

Each variant was a heterozygote and only counted if located in the same transcript of the gene. The GENE column contains the identified genes, the SAMPLE COUNT column contains the number of suicide samples in which a rare potentially damaging variant was found in the given genes.

*denotes genes without disease association in MalaCards and OMIM.

From these results, we can conclude that suicide is unlikely to be caused by a single dominantly inherited genetic allele.

### Identification of potentially recessive loss-of-function rare variants

In this study, no HI homo- or hemizygote (X-linked) variants were identified; except the ZSCAN1 gene, which had two HI variants in the same individual that complies with recessive inheritance. This gene encodes a zinc finger-domain transcription factor, which is expressed in the brain, but thus far has not been associated with any human disease (Table 4).

**Table 4:** Putative recessive loss of function candidate gene with two rare protein disruptive variants.

GENE	CHR	POS	REF	ALT	CONSEQUENCE	SAMPLE ID
ZSCAN1*	19	58549663	TC	T	frameshift variant	Y591
ZSCAN1*	19	58549495	G	A	stop-gain variant	Y591

CHR and POS show the GRCh37 coordinate of the variant; the REF/ALT columns shows the reference and alternate alleles; the CONSEQUENCE is the type of the protein disruption; the SAMPLE ID column contains the sample name in which the given variant was found in a heterozygote state.

*denotes gene without disease association in MalaCards and OMIM.

In the second part of this analysis, we also included MI variants in the study. We selected genes, where at least one suicide sample had an HI/MI variant in homo-, hemi- or compound heterozygous state. We excluded variants that were in the same allele, in the cases where we had phasing information. Only COL6A6 showed at least two heterozygote variants in three samples, all other genes had such variants in one sample only.

Among the 79 identified genes, 60 genes were on autosomal chromosome (Table S3). We found the following 12 genes, which were associated with neurological disorders: DNAH5, CTTNBP2, TSC2, NAV2, TG, PARD3B, CREB1, KCNB1, MAN1B1, NSD1, RERE, and ERCC5.

We also identified 19 X-linked candidate genes with hemizygous MI variants in male suicide individuals that fit to our criteria, but only one of these (RP2 gene) had been found in two male suicide victims (Table 5). We identified four genes that had already been associated with neurological disorders (AFF2, ALG13, OPHN1, and RBM10).

**Table 5:** Putative recessive loss of function X-linked candidate genes with hemizygous variants in male suicide victims.

GENE	CHR	POS	REF	ALT	CONSEQUENCE	SAMPLE ID
RP2	X	46696585	C	T	missense variant	Y558
RP2	X	46696543	G	C	missense variant	Y591
LINC00632*	X	139795826	G	A	missense variant	Y645
TSPAN6	X	99890198	C	T	missense variant	BrA206
PBDC1*	X	75397790	A	C	missense variant	Y482
NR0B1	X	30326933	C	A	missense variant	Y482
TIMP1	X	47444635	C	G	missense variant	BIB82
TFE3	X	48888013	C	T	missense variant	BrA206
TAB3*	X	30864180	A	G	missense variant	Y591
CXorf23*	X	19948685	G	A	missense variant	Y558
MAMLD1	X	149681090	A	C	missense variant	Y591
STARD8	X	67937097	A	G	missense variant	Y919
RBM10	X	47006890	G	A	missense variant	Y919
MAP3K15*	X	19398252	C	T	missense variant	Y645
ALG13	X	111003183	G	A	missense variant	Y787
ATP11C*	X	138884497	G	A	missense variant & splice region variant	Y166
TRO	X	54956296	G	A	missense variant	Y482
AFF2	X	148037417	G	T	missense variant	BIB82
DMD	X	31366742	C	T	missense variant	Y482
OPHN1	X	67273643	T	C	missense variant	BrA206

CHR and POS show the GRCh37 coordinate of the variant; the REF/ALT columns shows the reference and alternate alleles; the CONSEQUENCE is the type of the protein disruption; the SAMPLE ID column contains the sample name in which the given variants were found in heterozygote state.

*denotes gene without disease association in MalaCards and OMIM.

### Microsatellite analysis

STRs are composed of 2–6 bp DNA motifs that repeated approximately 5 to 50 times. STRViper and lobSTR analyses were carried out to investigate the potential role of these microsatellites within the exonic, intronic, and UTR sequences, as well as whether in the proximal promoter regions these could serve as genetic factors in suicide. In this analysis, we tested the hypothesis of whether the length of the repeat region may be an important factor. The LobSTR-based method is capable of analysing the short repeat sequences (<100 bp), where the reads fully contained the repeat. As a result of this approach, we could not detect significant differences in the lengths of STRs between the suicide and control groups. The STRviper approach is capable of analysing larger repeats^37^, but no single large STR expansion or contraction (as is the cause for example Huntington disease) were detected in any of the suicide samples. From this study, we can conclude that large STR-based variations do not support the monogenic disorder hypothesis. However, we mention here that longer repeats with minor variations cannot be examined with short-read sequencing.

### Analysis of copy number variation

The high coverage of sequencing reads allowed us to analyse the CNV polymorphism. In this part of the study, we investigated 286,754 high coverage (average coverage >20-fold) regions. Wilcoxon Rank-Sum test with Bonferroni multiple hypothesis testing correlation did not reveal any significant differences in the copy number of the investigated regions between control and suicide samples; thus, this kind of polymorphism alone is unlikely be responsible for suicidal behaviour.

### Gene network analysis

**Figure 1:**
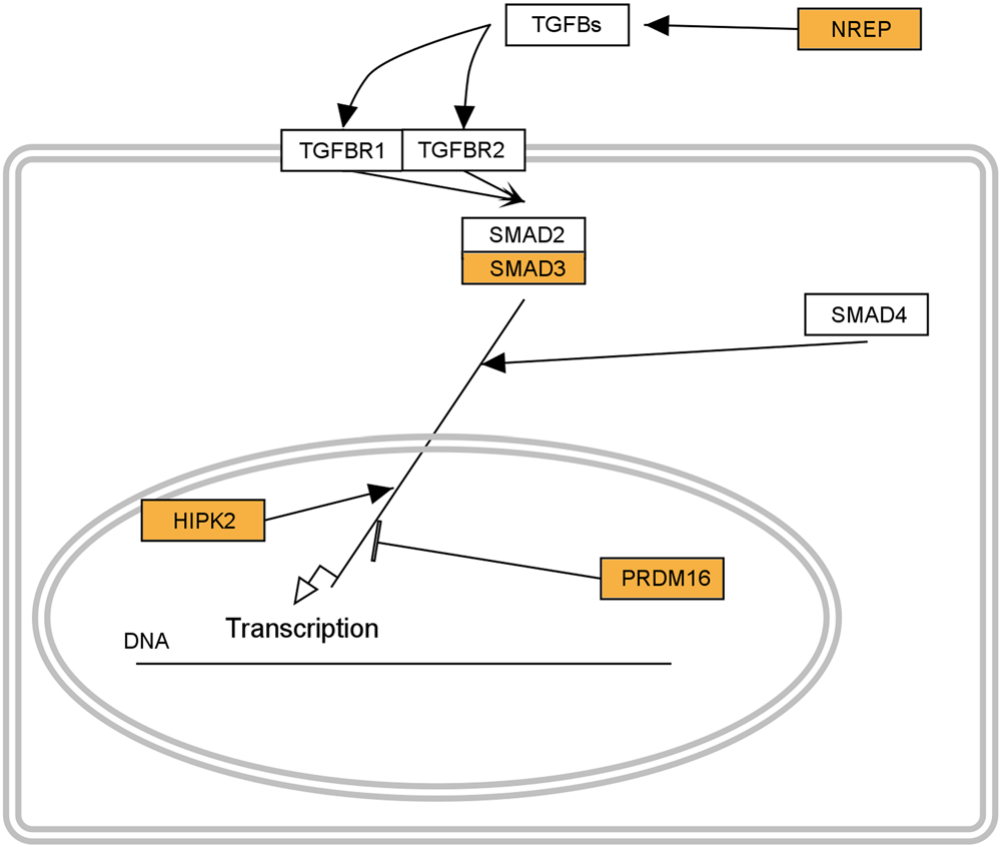
NREP, HIPK2, PRDM16, SMAD3 and their roles in the canonical TGF-β signalling pathway. The region-dependent accumulation of rare variants dataset was significantly enriched in the genes connected to the TGF-β pathway. Highlighted are the four genes contained in the dataset. Interactions were drawn using Pathvisio 3.2.4.

Of the 201 genes implicated by our analyses, 191 genes had an entry in the STRING database. 88 out of the 191 genes were interconnected; while 20 genes had interactions with experimental evidence forming a network as shown in Figs S1 and S2. The biggest hub in this network was SMAD3.The Enrichment Analysis function on the Gene Ontology (GO) website was used to identify gene sets from the obtained candidate genes, which cluster into gene networks. Four genes belonging to the transforming growth factor beta (TGF-β) signalling pathway have been found to be enriched (p = 0.0225; with α = 0.01 following Bonferroni correction) in the dataset obtained from the analysis of region-dependent accumulation of rare variants. Genes NREP, SMAD3, HIPK2 and PRDM16 are all members of the TGF-β pathway and the regulation of cellular response to TGF-β (Fig. 1). The SMAD3 is a transcriptional modulator, while the rest of the genes code for transcription factors. We have identified mutations in the 3′-UTRs of the SMAD3; HIPK2; and PRDM16 in 4–4 subjects each. Mutations of the NREP gene are accumulated in the splice region in six subjects. Note that SMAD3 is a hub protein with multiple interactions (Fig. S1), and that NREP directly acts on TGF-β.

Altogether, 15 suicide subjects had 18 variants, which may affect the function of the TGF-β pathway. The members of the TGF-β superfamily have been shown to play an important role in the formation of synapses and neural development^38^. The TGF-β1 itself was first implicated in the pathogenesis of depression when Myint and colleagues^39^ found significantly lower levels of this protein in the blood of patients diagnosed with MDD than in the control group. Since then numerous studies have confirmed the connection between MDD and TGF-β^40–43^. Studies were able to associate low levels of TGF-β1 with MDD but they have failed to associate TGF-β with suicidal behaviour. However, Lee and Kim^44^ examined attempted suicide, while O’Donovan and colleagues^45^ investigated suicide ideation, in contrast to our work, in which we investigated completed suicide. Furthermore, these reports examined the TGF-β itself, while our work detected four other genes of the TGF-β pathway as candidates for suicide. We did not obtain a significant enrichment of genes within a gene network for the rest of the datasets.

### Detection of viral DNA in the brain

We took advantage of the off-target reads and analysed them for the presence of virus sequences. We could detect human herpes virus 6 (HHV6) in the autopsy brain samples of suicide victims (HHV6B in two victims and HHV6A in one of the HHV6B-infected individuals); these sequences were not identified in the controls. The herpesviruses have been considered to play a role in the development of various neurological diseases including multiple sclerosis^46^, Parkinson’s disease, Alzheimer’s disease^47^ and epilepsy^48, 49^. However, due to the low sample size and frequency of infection, we cannot state for sure that HHV6 infection is a risk factor for suicide.

## Discussion

In this report, we performed WES analysis in brain samples of suicide victims, who suffered from MDD and control subjects who died from other causes. We searched for rare minor alleles found only in the suicide victims and not in our controls nor in the available human genome/exome databases. We also analysed the genes and intragenic sequences for potential multiple variants, the differences in the lengths of microsatellites and the CNV of the covered genomic regions. Additionally, gene network analysis of the candidate genes was also carried out. We also investigated the presence of potentially pathogenic viruses in the brain tissues. The effect of sampling bias was minimized by using gender-ratio-matched ethnic group (Hungarian) controls. In our suicide cohort, the male victims chose more extreme method of suicide (hanging or jumping; 14 out of 15 victims), while females chose less extreme means, namely drug overdose (5 out of 8 victims).

This genomic analysis focuses on the accumulation of genetic variants within the genes instead of individual variants. From our results, we can conclude that suicide is unlikely caused by a single gene, instead, it may be either an umbrella disease-like disorder caused by multiple high-penetrance genes, or it may be determined by the concerted action of multiple genes and the environment. We detected rare genetic variants, which potentially could be the causes of suicide. We emphasize the potential significance of the three calcium ion channel genes (CACNA1B, -1C, and -2D4), which we identified in two different analyses. The CACNA1A, another calcium channel gene, has also been proposed by others as a candidate gene for MDD^35^. This report also identified several candidate genes that have not been associated with any diseases until now. Additionally, our study also revealed that the TGF-β signalling pathway may play a causative role in the completed suicide (Fig. 1), which is a confirmation of earlier studies that proposed a role for TGF-β in MDD^40–44^. Members of the TGF-β superfamily have been shown to play an important role in the formation of synapses and neural development in invertebrates^50, 51^ and in vertebrates^38, 52^ as well. Alteration in TGF-β signalling may also shift the balance of pro-inflammatory and anti-inflammatory cytokines, as proposed in MDD^39^. However, since no other controls (non-suicide MDD patients and non-MDD suicide victims) were used in this study, the identified candidate genes and the TGF-β signalling pathway may be general genetic factors for the depression or for the suicide, instead of having potential exclusive roles for the completed suicide in MDD patients.

## Materials and Methods

### Clinical samples and ethical statements

Brains from suicide victims (n = 23; 15 males and eight female) and from control participants (n = 21; 14 males and seven females) were used for whole-exome sequencing. Samples were taken from the occipital cortex, cerebellar cortex and somatomotor cortex of individuals. All patients died suddenly from causes not directly involving any CNS diseases. Tissue samples were obtained by autopsy at the Department of Forensic Medicine of the Semmelweis University Medical School. In the case of suicide victims, a psychiatric diagnosis of MDD was on record. These were done and/or confirmed by experienced psychiatrists on the basis of criteria defined in the fourth edition of Diagnostic and Statistical Manual of Mental Disorders. Suicide victims died by hanging (n = 16), drug overdose (n = 6), or jump from height (n = 1). Causes of death in control subjects were the following: acute heart failure (n = 4), myocardial infarction (n = 6), cardiorespiratory insufficiency (n = 4), chronic hepatitis (n = 2), chronic bronchitis (n = 1), Alzheimer’s disease (n = 2), acute cardiopulmonary insufficiency (n = 1), and stroke (n = 1). Examination of medical records of control subjects at the autopsy confirmed the absence of psychiatric illness within the past 10 years. All of the controls and suicide victims were Caucasians of Hungarian ethnicity (Budapest region). Harvesting of tissues was approved by the local ethics committee^53^. Data were analysed anonymously.

### Whole exome sequencing

Whole-exome sequencing was carried out in DNAs of 23 suicide victims and 21 controls using *post mortem* brain tissues as a source. Genomic DNA samples were purified from the cortex regions using the DNeasy Blood and Tissue Kit (Qiagen) according to the manufacturer’s protocol. (30 mg tissue was used from each sample for the DNA extraction.) The qualified genomic DNA (200 ng from each sample) were fragmented by Covaris technology with resultant library fragments of 200–450 bp. Whole exome sequencing was performed as previously described (Chen *et al*., 2013) with slight modifications. In brief, whole exome enrichment was performed with the Agilent SureSelect Human All Exon V5+UTRs kit (Agilent Technologies, Santa Clara, CA), following the kit’s recommendations and sequenced with the Illumina HiSeq. 2000 sequencer (Illumina, San Diego, CA) to generate 100 bp-paired end reads. BWA MEM (version 0.7.9a-r786) was used to align reads to the GRCh37 reference genome. Genome Analysis Toolkit (GATK) HaplotypeCaller (version 3.5) best practices^54^ were used to generate final quality recalibrated BAM files for downstream analysis and variant calling. SnpEff^55^ (version 4.3) with GRCh37.75 data set was used for annotating variants. We obtained very high coverage per base position in both cohorts; in the Hungarian control cohort 96.3–97.5% (quartiles) of target regions had higher than 20-fold and 81.6–91.6% of target regions had higher than 40-fold coverage. In the suicide cohort these values were 95.4–97.5% for 20-fold and 82.8–90.5% for 40-fold coverage, respectively. We obtained on average ~130,000 variants per sample using the SureSelect V5 Plus all Exon kit for the exome sequencing. We identified 442,270 unique variants in the suicide and control cohorts altogether. In the study of biallelic variants, we excluded 11,634 multiallelic-, 2,344 pseudogenic-, and 6,234 refseq errors, as well as, 27,565 low complexity-, 32,025 uncertain-, and 68,015 low-depth variants. Among the remaining 294,453 high quality, biallelic variants 30,206 had no MAF values and 264,247 biallelic variants had known MAF values in the public (dbSNP and/or EVS) databases.

### Analysis of biallelic variants

To filter out high quality biallelic variants that follow HWE, we used the following criteria for the exclusion: variants failed PASS filter for >10% of samples; variants where coverage were <10x for >10% of samples; variants in low complexity repetitive regions were excluded (the analysis was based on the Dust score of flanking 15 bases of the reference sequence 5′ and 3′ around the variant); technical errors (wetlab or bioinformatic) and pseudogenic variants that violated HW equilibrium in controls. We defined common variants that had minor allele frequency (MAF) in dbSNP (build 146 GRCh37); ~6,000 exome data of Exome Variant Server (EVS); ~60,000 exome data of ExAC database; or it was found in more than two samples in Hungarian control group. We investigated rare variants with MAF < 1/5,000 values in the public databases (dbSNP, EVS, ExAC), assuming dominant, and MAF < 1/$\sqrt{\text{5,000}}$, assuming recessive Mendelian inheritance. The following two scenarios were tested: in the gain-of-function scenario, we tried to find putative regulatory or coding regions of the genes exhibiting region-specific accumulation of mutations in the suicide cohort but not in the control group; in the loss-of-function scenario, we assumed random distribution for the potentially protein-disrupting variants (high- or moderate-impact (MI) mutations) throughout the coding and UTR regions of the genes.

#### Analysis of rare variants - region dependent accumulation

For this analysis, we created a bed coordinate list based on the original SureSelect V5 All exon + UTR Plus target region that contained all 5′ UTR, 5′ flanking intronic, exonic, 3′ flanking intronic, and 3′ UTR regions separately. We investigated all regions searching for the accumulation of rare (AF < 1/5000) variants in the control and the suicide cohorts. Similarly to the approach that is used at ExAC^56^ for the classification of genes by the ratios of expected and observed synonymous and potentially damaging mutations, for each bed coordinate the count of rare variants was determined for both control and suicide cohorts. The analysed regions have been ranked on the basis of accumulation differences in rare variants between the control (expected) and suicide (observed) cohorts. Assuming random distribution of rare variants Monte Carlo simulation showed that the chance of having at least 4 rare variants (out of 14,393 rare variants in the suicide cohort) in any region (594,910 regions in total) is approximately 1%. Thus, a region with 4 or more rare variants observed in suicide cohort compared to the controls was considered a candidate.

#### Analysis of rare variants - Loss-of-function – putative dominant mutation

Based on SnpEff annotation of variants we selected rare variants that had HI or MI in any of the transcripts of genes covered in the exome kit (see SnpEff classification); those that had MAF < 1/5000 (assuming ~1:5000 incidence of suicide in Hungary) and more than two suicide patients had at least one of such variant in the same transcript. We also selected all HI variants in the cohort of suicide patients. From both lists we excluded bogus transcripts (no START codon, multiple STOP codons, incomplete transcript) and those transcripts that had any high- impact variant in public databases or in Hungarian controls that had MAF higher than 1/5000.

#### Analysis of Low-frequency variants - Loss-of-function – putative recessive mutation

Based on SnpEff annotation of variants we selected rare variants that had HI or MI in any of the transcripts of genes covered in the exome kit; had MAF < 1/71 (considering ~1:5000 incidence in Hungary, and recessive/homozygote/p^2^/frequency) and more than two suicide patients had at least two (or one homo- or hemizygote) of such variants in the same transcript. Furthermore, we excluded those variants that were located in the same allele, where we had phasing information (variant distance <100 bp read length). We selected transcripts which contained homozygote, hemizygote or compound heterozygote HI variants (compound heterozygosity: both alleles are mutated but at different locations). We also created a list where at least two HI heterozygote variants (or one homo-, hemizygote) were found in the same transcript in any of the suicide patients. From both lists we excluded bogus transcripts (no START codon, multiple STOP codons, incomplete transcript) and those transcripts that had any HI variant in public databases or in Hungarian controls that had MAF higher than 1/71.

### Microsatellite analysis

We used STRViper^37^ to identify possible large STR expansions or contractions and lobSTR (version 3.0.2) according to its best practice guides for exome data analysis^57^ to calculate exact STR counts of smaller repeats that were entirely spanned by the reads. STR counts of suicide and control were analysed by Wilcoxon Rank-Sum test with Bonferroni multiple hypothesis test correction. Wilcoxon-Mann-Whitney power analysis (alpha = 1.472e-4, SD = 1.07) showed that at the given sample size only large repeat differences (two or more repeats) between the two cohorts could be statistically analysed.

### Analysis of copy number variation

In this analysis, we first refined the target regions specified in the original SureSelect V5 All exon + UTR Plus target region to contain only the actual high coverage BED coordinates from the empirical sequence data of the control group using “samtools depth” algorithm (avg coverage >20x). “Samtools bedcov” algorithm has been applied on the high coverage BED coordinates identified in the previous step in order to calculate the coverage of sequencing reads for individual regions. Coverage data was normalised by the total GRCh37 mapped reads for each individuals and regions. Finally, matrix of 286,754 × 23 and 286,754 × 21 region/coverage data were obtained for the suicide and control groups, respectively. Due to the possibility of biological CNV variation, the more robust Wilcoxon-Mann-Whitney test has been applied for data and power analysis and to calculate the p values. Power analysis showed that at 0.95 power, alpha = 1.74e-7 (multiple hypothesis test correction to 286,754 regions), considering 50 percent coverage change between the two groups, 19 control and 19 suicide samples (Wilcoxon-Mann-Whitney test, with SD = 0.17) are needed to reveal significant differences.

### Gene network analysis

All the genes implicated by our analyses were considered for gene network analysis. The STRING database was used to search for experimentally proven interactions between our candidate genes. In order to determine whether genes connected to suicidal behaviour are significantly enriched in a gene network, we used the Enrichment Analysis function on the Gene Ontology (GO) website (www.geneontology.org)^58^, which combines the GO database with the PANTHER database^59^. We conducted separate searches for each of the four sets of genes (Table S4) identified as potential genetic factors for suicide. We also conducted an enrichment analysis containing all the genes in the four datasets.

### Detection of viruses in the brain

In this analysis, we extracted all the reads that were not mapped to human GRCh37 reference sequence in each sample from the corresponding BAM files. Using a custom script, we converted them to a multiline FASTA file where the QNAME (See SAM file specification) field was used as sequence ID and the sequence was extracted from each of the unmapped reads. Standalone BLAT^60^ was used to align the FASTA files of each sample to the NCBI curated all viral genomes (ftp://ftp.ncbi.nlm.nih.gov/refseq/release/viral/). BLAT hits with >90 bp length and >95% identity were filtered out and summarized for suicide and control cohorts using custom scripts.

### Data Availability

Sequenced data were deposited at the Sequence Read Archive (SRA) under BioProject SUB2335490).

## Electronic supplementary material

Supplementary Figures (1 and 2)

Supplementary Table 1

Supplementary Table 2

Supplementary Table 3

Supplementary Table 4
