# Supplementary material for: High-Coverage Whole-Exome Sequencing Identifies Candidate Genes for Suicide in Victims with Major Depressive Disorder
Source: Sci Rep. 2017 Aug 2;7:7106. doi: 10.1038/s41598-017-06522-3 (PMC5541090; doi:10.1038/s41598-017-06522-3)
Supplement: Supplementary file 1 — Supplementary Figures (1 and 2) [file 41598_2017_6522_MOESM1_ESM.pdf]

High-Coverage Whole-Exome Sequencing Identifies Candidate Genes for Suicide in Victims with Major Depressive Disorder

Dóra Tombácz[1,2,†], Zoltán Maróti[3,†], Tibor Kalmár[3], Zsolt Csabai[1], Zsolt Balázs[1], Shinichi Takahashi[2], Miklós Palkovits[4], Michael Snyder[2*], Zsolt Boldogkői[1*]

Addresses:

[1]Department of Medical Biology, Faculty of Medicine, University of Szeged, Somogyi B. u. 4., Szeged, H-6720, Hungary

[2]Department of Genetics, School of Medicine, Stanford University, 300 Pasteur Dr., Stanford, CA 94305-5120, USA

[3]Department of Paediatrics, Faculty of Medicine, University of Szeged, Somogyi B. u. 4., Szeged, H-6720, Hungary

[4]Neuromorphological and Neuroendocrine Research Laboratory, Department of Anatomy, Histology and Embryology, Semmelweis University, Budapest, Üllői u. 26., H-1085, Hungary

[†]These authors contributed equally to this work.

[*]Co-corresponding authors

E-mails:

tombacz.dora@med.u-szeged.hu

maroti.zoltan@med.u-szeged.hu

kalmar.tibor@med.u-szeged.hu

csabai.zsolt@med.u-szeged.hu

balazs.zsolt@med.u-szeged.hu

palkovits.miklos@med.semmelweis-univ.hu

takahashi.shinichi.dz@daiichisankyo.co.jp

mpsnyder@stanford.edu

boldogkoi.zsolt@med.u-szeged.hu

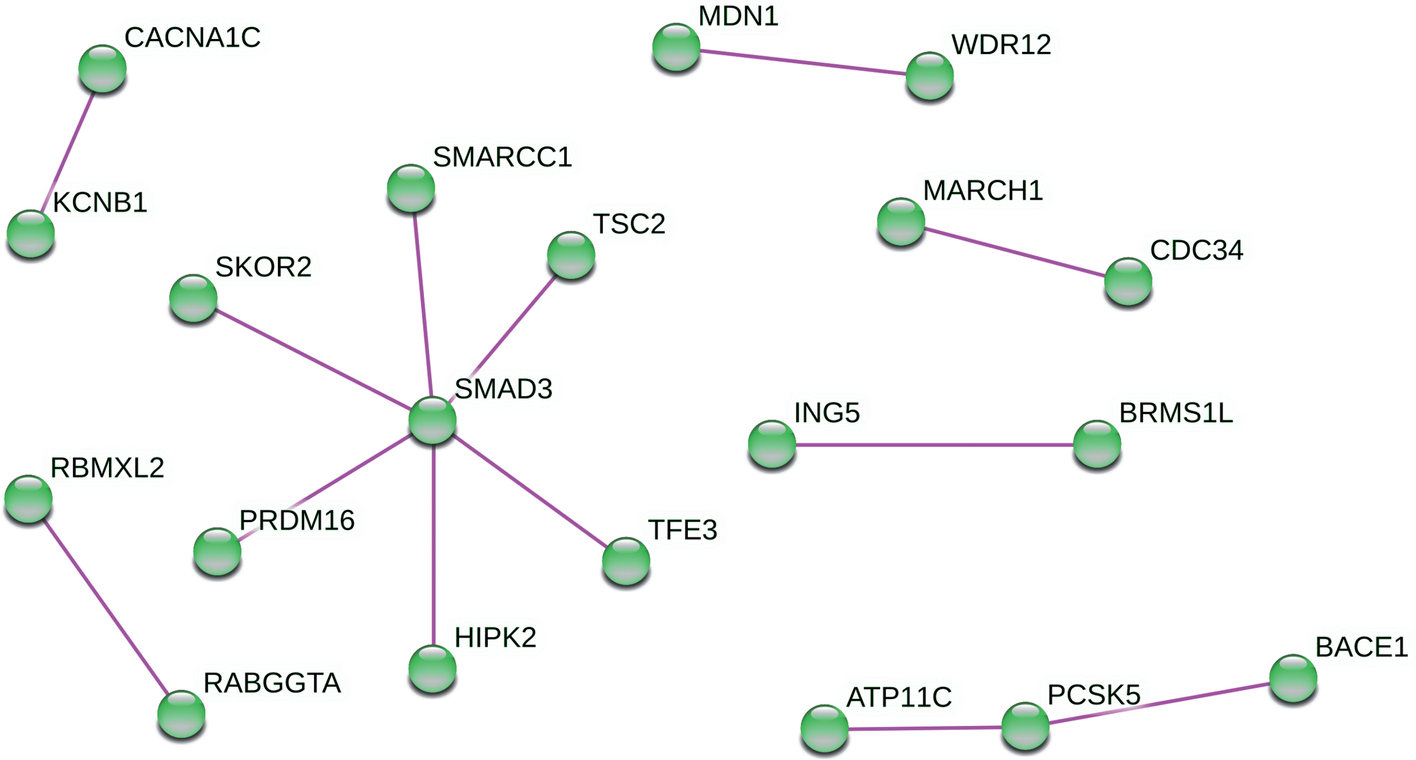

S1 Figure. Gene network of candidate genes based on experimental evidence of interaction. The gene network was constructed from all the implicated genes (S4 Table) using the string database accessible at http://string-db.org/ (version 10.5). All edges represent experimental evidence of interaction. Confidence cut-off was set to 0.333.

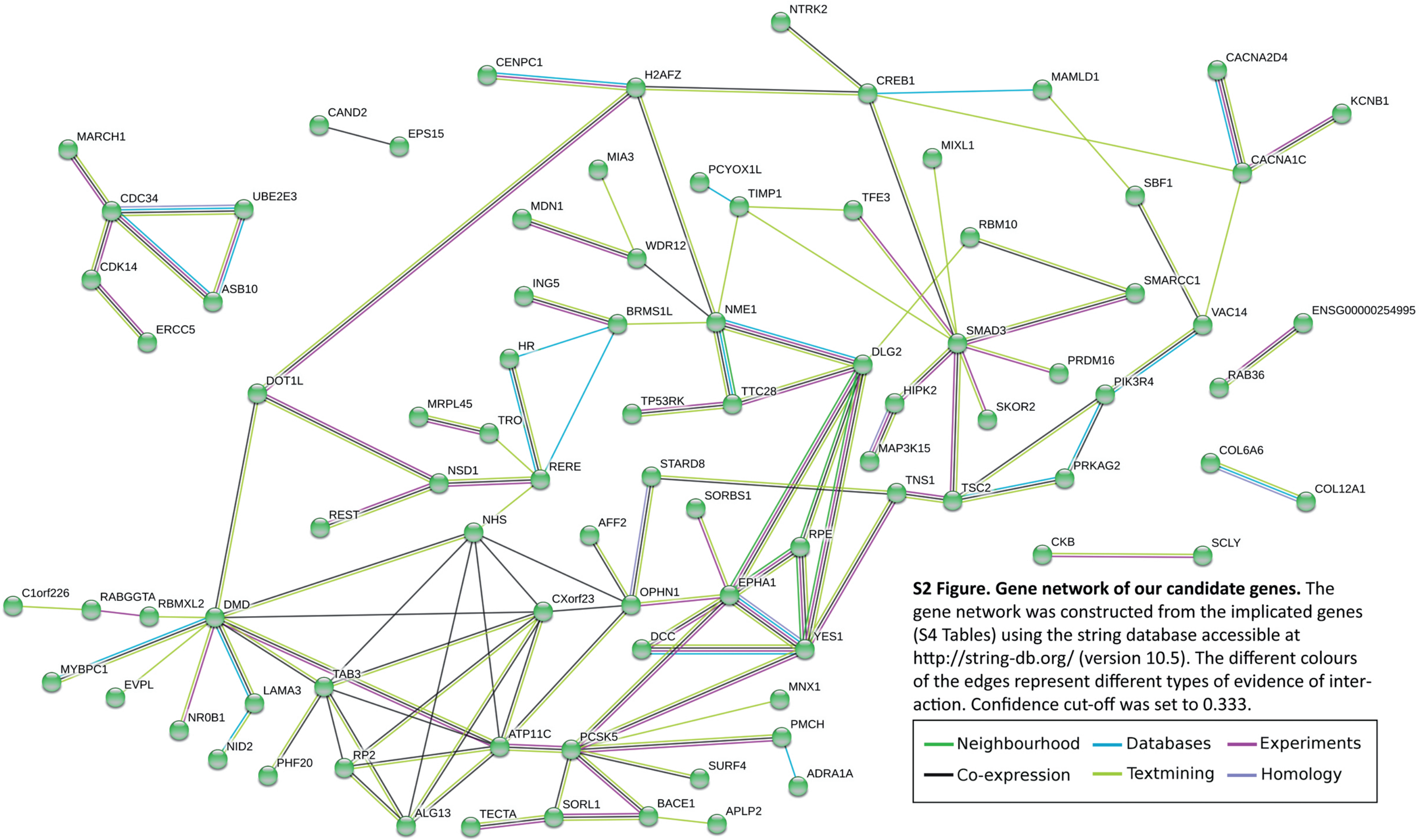

S2 Figure. Gene network of our candidate genes. The gene network was constructed from the implicated genes (S4 Tables) using the string database accessible at http://string-db.org/ (version 10.5). The different colours of the edges represent different types of evidence of interaction. Confidence cut-off was set to 0.333.
